# NIR-Fluorescent Hybrid Materials of Tm^3+^ Complexes Carried by Nano-SiO_2_ via Improved Sol–Gel Method

**DOI:** 10.3390/nano10101964

**Published:** 2020-10-03

**Authors:** Yanxin Wang, Qiuyu Sun, Linjun Huang, Peng Lu, Xiaozhen Wang, Zhe Zhang, Yao Wang, Jianguo Tang, Laurence A. Belfiore

**Affiliations:** 1 Institute of Hybrid Materials, National Center of International Joint Research for Hybrid Materials Technology, National Base of International Sci. & Tech. Cooperation on Hybrid Materials, College of Materials Science and Engineering, Qingdao University, 308 Ningxia Road, Qingdao 266071, China; SQY17861431023@163.com (Q.S.); 18753360989@163.com (P.L.); wangxiaozhen686868@163.com (X.W.); 18363995370@163.com (Z.Z.); wangyaoqdu@126.com (Y.W.); 2 Department of Chemical and Biological Engineering, Colorado State University, Fort Collins, CO 80523, USA; laurence.belfiore@colostate.edu

**Keywords:** NIR fluorescence, nano-SiO_2_, thulium complex, hybrid materials

## Abstract

Tm^3+^ has obvious emission characteristics in the near-infrared band. Thulium ions combined with different organic ligands lead to different fluorescent properties. In the near-infrared region, Tm^3+^ is a down-conversion fluorescent material that is unstable under high temperature and acidic conditions. Moreover, in those complex environments, the fluorescence from Tm^3+^ complex is usually degraded. In this work, two kinds of near-infrared fluorescent complexes, Tm(TTA)_3_phen and Tm(DBM)_3_phen, were prepared, and the intensity of their fluorescence is compared. The fluorescence intensity at 802 nm is greatly improved compared with Tm(TTA)_3_phen, and the intensity of the emission at 1235 nm and 1400–1500 nm is also enhanced. Moreover, the emission lifetime of SiO_2_-Tm(TTA)_3_phen is 50.38 μs. Tm(TTA)_3_phen complex and SiO_2_-Tm(TTA)_3_phen hybrid materials have better fluorescence than Tm(DBM)_3_phen and SiO_2_-Tm(DBM)_3_phen. Therefore, HTTA is a better choice of organic ligands for Tm^3+^. The NIR-fluorescent hybrid materials prepared have stronger fluorescence after combining with nano-SiO_2_compared with pure Tm^3+^ complexes, and have stronger structural stability compared with pure nano-SiO_2_.

## 1. Introduction

Rare earth ions complexes have special physical and chemical properties, which have attracted much attention thanks to their high purity, narrow emissivity, and high internal quantum efficiency. The fluorescent properties and mechanism of rare earth ion complexes have been studied for a long time. As early as 1942, Weissman investigated the fluorescence effect of β-diketone ligands on europium ions by ultraviolet radiation, and further studied the fluorescence efficiency of the complex [1,2,3,4]. At present, many organic ligands with large absorption cross sections in ultraviolet region have been found. The energy of the excited state can be transferred to the rare earth ions through the energy transfer inside these organic ligands, so the fluorescence intensity of rare earth ions can be greatly improved. Therefore, the rare earth ions fluorescent complexes formed by the combination of rare earth and organic ligand have excellent fluorescence [5,6,7]. However, the fluorescence properties of rare earth complexes can be damaged in complex environments. In the preparation of many fluorescent materials, rare earth ions cannot be prepared at a high temperature, and the solution concentration should be controlled. Owing to the large amount of coordination with rare earth ions, complexes are easy to coordinate with solution molecules, resulting in fluorescence quenching, and a decrease of the thermal stability. Therefore, high transparency, good rigidity, and three-dimensional network void structure of nano-SiO_2_ is chosen as the matrix, which can not only protect the stability of rare earth ions complexes, but also keep or increase the fluorescence of hybrid materials.

Therefore, the hybrid material prepared by the rare earth complex and nano-SiO_2_ is a promising strategy to protect the rare earth complex, improve their thermal stability, and to be used to prepare durable functional materials [6]. In order to be used in different fields, there has been a wealth of research on controlling the size of nano-SiO_2_. Rao et al. prepared nano-SiO_2_ with the size of 20–460 nm by the simple sol–gel method [8]. Rahman et al. found that, in the presence of a small amount of NH_4_Br, a single size of 20–34 nm could be synthesized [9]. Kim et al. reported that the introduction of dielectric could reduce the size of nano-SiO_2_ to 17.5 nm [10]. However, it is difficult to give consideration to both morphology and fluorescence because of the special amorphous structure of nano-SiO_2_, so there are few reports on the rare earth doped silica materials with special morphology [11].

By introducing different templates into the sol–gel method, the micro-morphology of SiO_2_ can be controlled. Among them, one template mainly includes surfactants, organic acids, and gel factors [12,13]. Another template mainly includes porous alumina film and insoluble helical polymer. For example, using dodecyl trimethylammonium bromide (CTAB) as a template, spherical and short rod-shaped nano-SiO_2_ materials were obtained by adjusting the amount of water. Moreover, different tartaric acid derivatives were used as templates to synthesize nano-SiO_2_ hollow microspheres, SiO_2_ nanoribbons, and SiO_2_ nanotubes [14,15,16]. Compared with previous reports, in order to reduce the influence of H_2_O on the fluorescence of Tm(TTA/DBM)_3_phen, we have improved the experimental method for preparing nano-SiO_2_, and changed the size of nano-SiO_2_ by adjusting the dosage of NH_3_·H_2_O.

Here, we report the synthesis of SiO_2_-Tm^3+^(SiO_2_-Tm(TTA)_3_phen and SiO_2_-Tm(DBM)_3_phen) hybrid materials via an improved method, which not only embeds Tm^3+^ complexes inside nano-SiO_2_, but also embeds a part of Tm^3+^ complexes on the surface of nano-SiO_2_. Thus, Tm^3+^ complexes (Tm(TTA)_3_phen, Tm(DBM)_3_phen) can be more richly filled in SiO_2_, and the fluorescence and stability of Tm^3+^ complexes can be enhanced.

Like ordinary electromagnetic waves, infrared light has wave–particle duality. The energy of infrared light is exactly equal to the energy difference between different energy states of molecules, so infrared absorption effect occurs. Because of the differences in fat content, sugar content, and freshness of food, as well as the differences in biological tissues, there are also differences in the absorption of infrared light waves in food and biological tissues. According to the differences in absorption, different food and biological tissues can be detected. Therefore, infrared fluorescent materials with good fluorescence properties provide favorable conditions for the development of biomedical and food detection.

## 2. Experimental and Characterization

### 2.1. Materials

Tetraethoxysilane (TEOS), 4,4,4-trifluoro-1-2-thenoyl-1,3-butanedione (HTTA), dibenzoylmethane (HDBM), ammonium hydroxide (NH_3_·H_2_O, 98%, AR), and thulium oxide (Tm_2_O_3_, 99.99%, AR) were purchased from Hu Shi Chemical Plant (Shanghai, China). 1,10-Phenanthrolinemonohydrate (Phen, 99%, AR) was purchased from China National Medicines Group (Beijing, China). TmCl_3_ ethanol solution (EtOH) was prepared as follows: Tm_2_O_3_ was dissolved in concentrated hydrochloric acid (HCl), and the surplus HCl was removed by evaporation. The residue was dissolved in anhydrous ethanol.

### 2.2. Synthesis of Tm^3+^ Complex

The Tm^3+^ complex (Tm(TTA)_3_phen, Tm(DBM)_3_phen)was prepared according to the following process. HTTA/HDBM and Phen in a stoichiometric molar ratio were dissolved in a suitable volume of anhydrous ethanol. The mass of HTTA/HDBM and Phen was 1.332 g/1.447 g and 0.396 g respectively, which were mixed, heated, and stirred for 1 h in a beaker. Then, an appropriate amount of 20 μL sodium hydroxide solution was added to the solution. A stoichiometric amount of TmCl3 ethanol solution was then added dropwise to the solution while stirring. The stirring temperature was 60 °C. The molar ratio of Tm^3+^/HTTA/HDBM/Phen was 1:3:1. Then, the pure complexes were collected by centrifugation and alcohol washing for three times. The final products were dried in oven at 50 °C and white powder was obtained.

### 2.3. Synthesis of SiO_2_-Tm^3+^ Hybrid Materials

An improved sol–gel process was employed in this work. Firstly, HTTA/HDBM and Phen were dissolved in a certain volume of anhydrous ethanol according to the stoichiometric molar ratio. The mass of HTTA/HDBM and Phen was 1.332 g/1.447 g and 0.396 g, respectively, and the solution was magnetically stirred for 0.5 h at 60 °C at 700 r/min. Next, 3 mL of NH_3_·H_2_O was added to 50 mL of ethanol. After 30 min, 3 mL TEOS was added to the above solution, then we adjusted the dosage of NH_3_·H_2_O, ethanol, and TEOS. Compared with previous research, no more water was added. The preparing method was improved and it saved water resources. Then, the proper amounts of silica gel solution, Tm(TTA/DBM)_3_phen solution, and Tm(TTA/DBM)_3_phen powder were mixed, heated, and stirred for 3 h, and then centrifuged (speed: 10,000 r/min, 10 min). The excessive Tm(TTA)_3_phen was removed by three times extensive washing with ethanol. The final products were dried in oven at 50 °C and white powder was obtained.

### 2.4. Characterization

A JEOL JEM-2100F transmission electron microscope was used for the identification of morphology and size of hybrid nanoparticles. The structure and crystal phases of Tm(TTA)_3_phen/Tm(DBM)_3_phen and SiO_2_-Tm(TTA)_3_phen/Tm(DBM)_3_phenwere determined by powder X-ray diffraction (XRD, Ultima IV, Rigaku Corporation, Japan). FT-IR of Tm(TTA)_3_phen/Tm(DBM)_3_phen, SiO_2_-Tm(TTA)_3_phen/Tm(DBM)_3_phen and SiO_2_ were performed using Nicolet5700. The elemental mapping was determined using an FEI (Field Electron and Ion Co.,) Talos F200i microscope (Thermo Fisher Scientific Inc., Waltham, MA, USA) operated at 200 KV. UV–vis absorption spectra were recorded on a Youke UV-755B spectrophotometer. The elemental composition was determined using scanning transmission electron microscopy with energy-dispersive X-ray spectroscopy (STEM-EDS), using an FEI Tecnai G2 F20 S-TWIN (FEI Inc., Hillsboro, OR, USA). Fluorescence spectra were measured by Edinburgh FLS-1000 steady-state transient fluorescence spectrometer (Edinburgh Inc., Livingston, UK) (342W Xe lamp).

## 3. Results and Discussions

**Figure 1:**
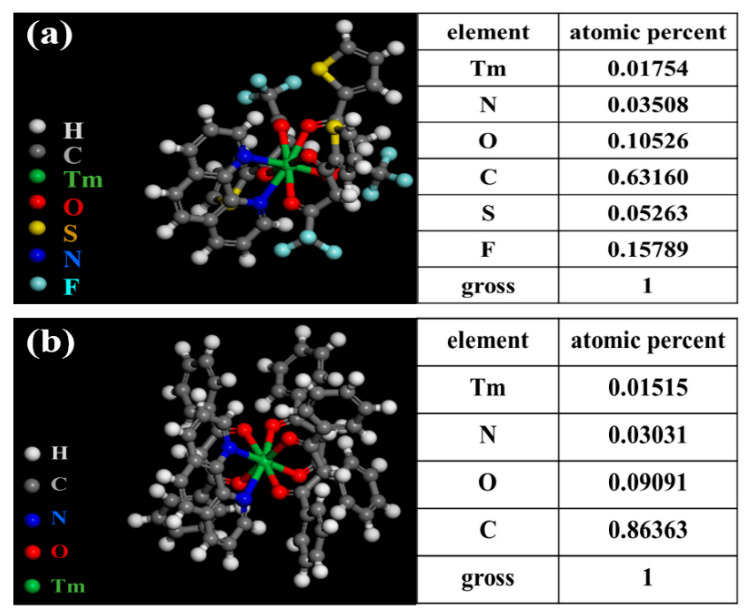
Predicted structure and EDS of (**a**) Tm(TTA)_3_phen; (**b**) Tm(DBM)_3_phen.

The structure and composition of Tm(TTA)_3_phen were investigated by EDS (Energy dispersive spectrometer) analysis. Figure 1a shows the predicted structure of Tm(TTA)_3_phen and the EDS clearly shows that S, F, N, O, C, and thulium elements are present in the Tm(TTA)_3_phen. According to the element composition obtained by EDS, F and S elements belong to HTTA, while N elements belong to Phen. By calculating the elements content of F, S, N, and thulium, we obtain the ratio between different groups to be Tm^3+^/HTTA/Phen = 1:3:1 [10]. The structure and composition of Tm(DBM)_3_phen are similar to Tm(TTA)_3_phen. N elements belong to Phen and O elements belong to HDBM. By calculating the elements content of C, N, O, and thulium, we obtain the ratio Tm^3+^/DBM/Phen = 1:3:1 [17].

**Figure 2:**
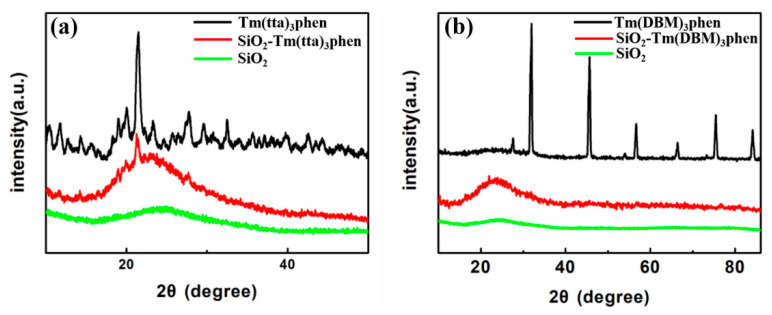
X-ray diffraction (XRD) patterns of (**a**) Tm(TTA)_3_phen, SiO_2_-Tm(TTA)_3_phen, and Nano-SiO_2_; (**b**) Tm(DBM)_3_phen, SiO_2_-Tm(DBM)_3_phen, and nano-SiO_2_.

Figure 2 shows the XRD of SiO_2_, Tm(TTA/DBM)_3_phen, and SiO_2_-Tm(TTA/DBM)_3_phen. Both SiO_2_-Tm(TTA)_3_phen and SiO_2_-Tm(DBM)_3_phen have a wide diffraction peak at 2θ = 20~25 in Figure 2a,b, which can be assigned to the amorphous silica, and the diffraction peak coincides with the characteristic peak of PDF#47-0715, without significant impurities. It is found that the diffracted intensity of SiO_2_ in SiO_2_-Tm(TTA/DBM)_3_phen hybrid materials is significantly higher than that of pure nano-silica. This is because Tm(TTA/DBM)_3_phen is embedded in the nano-SiO_2_, so the bond angle and bond length of SiO_2_ are changed, which improves the structural symmetry of nano-SiO_2_. Therefore, the hybrid materials have better structural stability. However, the diffraction peak of nano-SiO_2_ in SiO_2_-Tm(TTA)_3_phen is obviously stronger than that in SiO_2_-Tm(DBM)_3_phen, thus the structural stability of SiO_2_-Tm(TTA)_3_phen nano hybrid materials is stronger than that of SiO_2_-Tm(DBM)_3_phen. Furthermore, in Figure 2a, at the diffraction angle of 2θ = 21.731 and 2θ = 28.337, both of Tm(TTA)_3_phen and SiO_2_-Tm(TTA)_3_phen show a sharp and strong crystallization peak, which indicates that both of Tm(TTA)_3_phen and SiO_2_-Tm(TTA)_3_phen nanoparticles have high crystallinity. Figure 2b indicates that both Tm(DBM)_3_phen and SiO_2_-Tm(DBM)_3_phen nanoparticles have low crystallinity. Therefore, in terms of structure, the structural stability and composite degree of SiO_2_-Tm(TTA)_3_phen are obviously stronger than that of SiO_2_-Tm(DBM)_3_phen.

**Figure 3:**
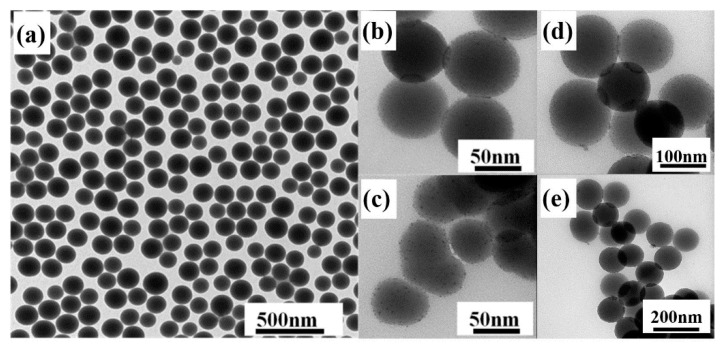
Transmission electron microscopy (TEM) image of (**a**) SiO_2_ nanoparticles; (**b**,**d**) SiO_2_-Tm(TTA)_3_phen; (**c**,**e**) SiO_2_-Tm(DBM)_3_phen.

**Figure 4:**
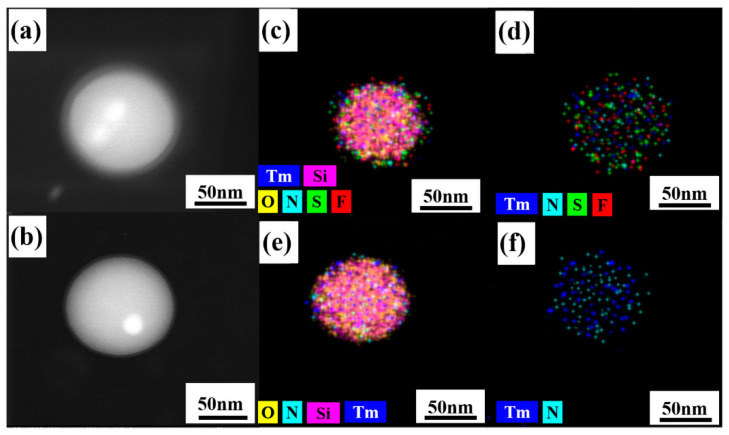
Scanning transmission electron microscopy (STEM) dark-field (DF) image of (**a**) SiO_2_-Tm(TTA)_3_phen and (**b**) SiO_2_-Tm(DBM)_3_phen; (**c**,**d**) elemental mapping of Si, O F, S, Tm, and N elements of SiO_2_-Tm(TTA)_3_phen; (**e**,**f**) elemental mapping of Tm, Si, O, and N elements of SiO_2_-Tm(DBM)_3_phen.

The structure and morphology of the samples were examined by TEM. From Figure 3a, we can see that the nano hybrid materials prepared by the improved method have good uniformity and dispersion in shape and size. The surface of nano-SiO_2_ is smooth and the nanospheres have a diameter of 80 nm. It is clear from Figure 3b–e that Tm^3+^ complexes were successfully attached to the surface of nano-SiO_2_ and some of Tm^3+^ complexes were embedded into SiO_2_. Figure 4c–f show elemental mapping of Si (pink), O (yellow), F (red), S (green), Tm (blue), and N (wathet). The existence of S, Tm, F, and N confirms the presence of the Tm(TTA/DBM)_3_phen complexes in the nano-SiO_2_.

According to TEM and XRD characterization, it can be concluded that SiO_2_ in SiO_2_-Tm(TTA)_3_phen hybrid materials provides better protection for Tm(TTA)_3_phen;combined with the TGA (Thermo Gravimetric Analysis) and fluorescence spectra, it can be concluded that the SiO_2_-Tm(TTA)_3_phen has better structural stability and better fluorescent performance than SiO_2_-Tm(DBM)_3_phen.

**Figure 5:**
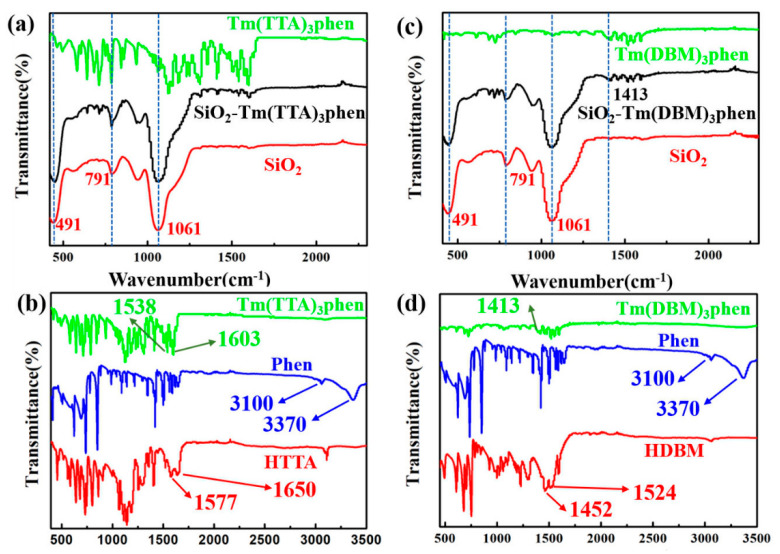
FT-IR spectra of (**a**) SiO_2_-Tm(TTA)_3_phen, Tm(TTA)_3_phen, and nano-SiO_2_; (**b**) Tm(TTA)_3_phen, Phen, and 4,4,4-trifluoro-1-2-thenoyl-1,3-butanedione (HTTA); (**c**) SiO_2_-Tm(DBM)_3_phen, Tm(DBM)_3_phen, and nano-SiO_2_; (**d**) Tm(DBM)_3_phen, Phen, and dibenzoylmethane (HDBM).

Figure 5 shows the FT-IR spectra of the SiO_2_-Tm(TTA/DBM)_3_phen hybrid materials, Tm(TTA/DBM)_3_phen complexes, and nano-SiO_2_. Figure 5a displays the FT-IR spectra of the Tm(TTA)_3_phen and SiO_2_-Tm^3+^ hybrid nanoparticles and SiO_2_. Obviously, the absorption peak of 1061 cm^−1^ is due to the frequency band of Si-O-Si symmetric telescopic vibration, and 491 cm^−1^ corresponds to the band of bending vibration of Tm-O-Si. The absorption peak at 791 cm^−1^ is the bending vibration peak of Si-OH. FT-IR spectra indicate that Tm(TTA)_3_phen and SiO_2_ combine to a hybrid material with a stable structure. Figure 5b shows the FT-IR spectra of the HTTA, Phen, and Tm(TTA)_3_phen. For Tm(TTA)_3_phen, the characteristic bands at 1577 cm^−1^ and 1656 cm^−1^ of HTTA disappear. At the same time, a strong absorption peak (C=O) appears at 1603 cm^−1^, indicating that the carbonyl group in HTTA is coordinated with Tm^3+^, and HTTA is confirmed as a non-negative bidentate ligand to coordinate with Tm^3+^. The coordination bond between Tm^3+^ and Phen is also formed in Tm(TTA)_3_phen. The absorption peak (C=N) of Phen appears around 1538 cm^−1^. The absorption peaks (N-H) at 3370 cm^−1^ and 3100 cm^−1^ in Phen disappear and form new coordination bonds with thulium ions. This indicates that the coordination between the two carbon atoms of Phen and thulium ions is bidentate coordination. FT-IR spectra indicate that Tm(TTA)_3_phen and SiO_2_ combine to form a hybrid material with a stable structure [18]. Similarly, when Tm(DBM)_3_phen is successfully combined with SiO_2_ in Figure 5c,d, the absorption peaks (N-H) at 3370 cm^−1^ and 3100 cm^−1^ in Phen disappear and form new coordination bonds with thulium ions. The stretching vibration of C=N originally located at 1413 cm^−1^ is red shifted, indicating that C=N is weakened to a certain extent. C=N and nano silicon hydroxyl on the surface of silicon are bonded by hydrogen to provide a site for the Tm^3+^ complex, while its strength is weakened. The absorption peak at 791 cm^−1^ is the bending vibration peak of Si-OH, while the peak at 491 cm^−1^corresponds to the band of bending vibration of Tm-O-Si [18].

**Figure 6:**
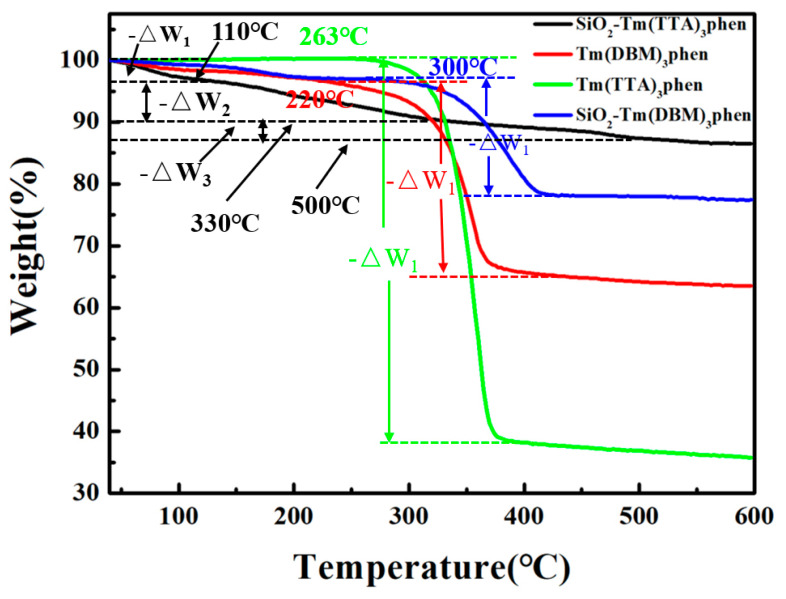
TGA curves of SiO_2_-Tm(DBM)_3_phen, SiO_2_-Tm(TTA)_3_phen, Tm(TTA)_3_phen, and Tm(DBM)_3_phen in nitrogen atmosphere(10 °C/min).

Figure 6 shows the TGA curves of SiO_2_-Tm(DBM)_3_phen, SiO_2_-Tm(TTA)_3_phen, Tm(TTA)_3_phen, and Tm(DBM)_3_phen, respectively. By thermo gravimetric analysis of SiO_2_-Tm(DBM)_3_phen, SiO_2_-Tm(TTA)_3_phen, Tm(TTA)_3_phen, and Tm(DBM)_3_phen, the thermal stability of the Tm(TTA/DBM)_3_phen and the SiO_2_-Tm(TTA/DBM)_3_phen was obtained, respectively. As can be seen from Figure 6, the thermal decomposition of Tm(TTA)_3_phen complexes shows 60% weight loss in the range of 263 °C to 380 °C, which is analyzed as the decomposition of two organic ligands in the Tm(TTA)_3_phen. After 400 °C, all the complexes decompose to thulium oxide. In addition, the thermal decomposition of SiO_2_-Tm(TTA)_3_phen is divided into three stages. In the first stage, there is 3% weight loss in the range of 40 °C to 100 °C, which is due to the removal of the adsorbed water on the surface of SiO_2_-Tm(TTA)_3_phen nanosphere. In the second stage, there is 7% weight loss in the range of 110 °C to 330 °C, which is due to the dehydration of water of colloidal nano-SiO_2_ in SiO_2_-Tm(TTA)_3_phen molecule. In the third stage, there is 3% weight loss in the range of 330 °C to 500 °C, which is due to the dehydration of crystal water in SiO_2_-Tm(TTA)_3_phen molecules. Thus, it can be seen that the thermal stability of Tm(TTA)_3_phen is greatly improved after combination with nano-SiO_2_. Moreover, by analyzing the thermal weight of SiO_2_-Tm(DBM)_3_phen and Tm(DBM)_3_phen, we can see that the initial decomposition temperature of the two nanomaterials is 220 °C and 300 °C, respectively. Therefore, the thermal stability of SiO_2_-Tm(DBM)_3_phen and Tm(DBM)_3_phen is less stable than SiO_2_-Tm(TTA)_3_phen; this is consistent with the results of TEM images analysis.

**Figure 7:**
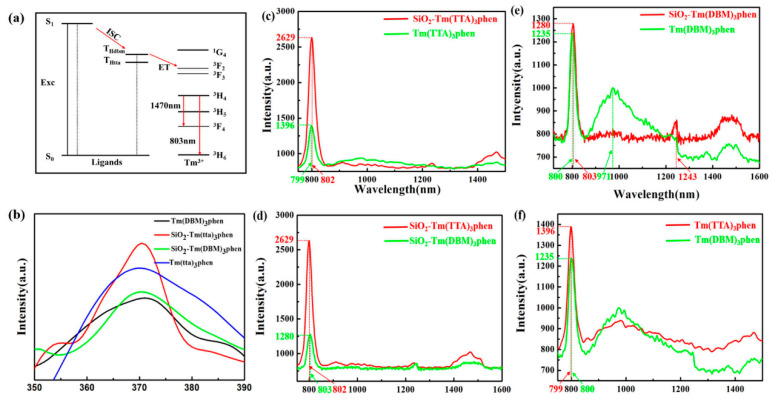
(**a**) Schematic energy diagram for the indirect excitation mechanism of theTm^3+^-ligands system; (**b**) excitation spectra of Tm(DBM)_3_phen, SiO_2_-Tm(TTA)_3_phen, SiO_2_-Tm(DBM)_3_phen, and Tm(TTA)_3_phen (λ_em_ = 802 nm). Fluorescent spectra of (**c**) Tm(TTA)_3_phen and SiO_2_-Tm(TTA)_3_phen, (**d**) SiO_2_-Tm(DBM)_3_phenand SiO_2_-Tm(TTA)_3_phen, (**e**) Tm(DBM)_3_phen and SiO_2_-Tm(DBM)_3_phen, and (**f**) Tm(TTA)_3_phen and Tm(DBM)_3_phen (λ_ex_ = 370 nm) (solid samples, λ_ex_ = 370 nm, slit width: 5 × 2 nm, liquid nitrogen refrigeration temperature: −80 °C).

Figure 7 shows the Jablonski diagram of prepared samples. The intra-molecular energy transfer mechanism shown in Figure 7a is the energy transfer process in Tm^3+^ complexes, which most scientists agree with Crosby’s theory [19] on the energy transfer mechanism of organic ligands transferring to Tm^3+^. In this process, Tm^3+^ is no longer first excited by the external excitation energy, but the organicligands of the Tm^3+^ complex first absorb energy, which is excited from the ground state S_0_ to the first excited state S_1_. Then, the molecules are internally transformed to the low-excited single state S_1_. In the next step of the process, the energy is transferred from the lowest excited singlet S_1_ to the excited triplet T_1_. Next, the energy is transferred from the lowest excited triplet T_1_ to the Tm^3+^ by virtue of the chemical bond of the Tm^3+^ complex itself. Thulium ion receives energy and is stimulated to produce an energy level transition, emitting characteristic fluorescence as they return to the ground state. If the energy level of the lowest excited triplet (T_1_) is lower than that of the excited state of rare earth ion, effective energy transfer cannot occur [20]. Recursively, the effect of this organic ligand′s energy transfer to the fluorescence material central ion is called the “antenna effect” [21,22]. Because of the stronger absorption capacity of HTTA in ultraviolet region and higher excitation state energy transfer efficiency than HDBM, HTTA helps to shorten the transition state of thulium ion and enhance the absorption coefficient of thulium ion in ultraviolet region. As the absorption coefficient of lanthanide ions is extremely low, and f-f level transitions of rare earth ions are forbidden, the ions must be combined with appropriate ligands. In this case, the excitation energy generated by the ligands absorbing photons in the ultraviolet region is transferred to the 4f resonance level [23]. Basically, intra-molecular energy transfer in thulium ion complex molecule is strongly affected by the energy difference between T_1_ of donor and 4f electronic levels of thulium ions acceptor [24,25,26]. In the energy transfer process from organic ligands to thulium ion, the optimal energy difference is conducive to effective energy transfer. TheT_1_ of HTTA and HDBM are 20,400 cm^−1^ and 21,700 cm^−1^, respectively. Moreover, the energy difference between the ^3^F_2_ level of HTTA and thulium ion is 7900 cm^−1^ [27], which is obviously lower than the energy difference between the ^3^F_2_ level of HDBM and thulium ion. Therefore, Tm(TTA)_3_phen will have stronger fluorescence than that of Tm(DBM)_3_phen.

The excitation spectra of Tm^3+^ complexes and SiO_2_-Tm^3+^ hybrid materials are shown in Figure 7b. The excitation spectra of two thulium ions complexes containing different organic ligands have wide absorption bands, which are mainly attributed to the π–π* electron transfer of the organic ligands. However, the absorption band of HTTA is significantly stronger than that of HDBM. In addition, the fluorescence sensitization of HTTA is much more effective than that of HDBM. The excitation spectrum of SiO_2_-Tm(TTA)_3_phen is narrower and stronger than that of Tm(TTA)_3_phen, Tm(DBM)_3_phen, and SiO_2_-Tm(DBM)_3_phen, but the location of the excitation peak and the absorption band position of the organic ligands remain unchanged. This suggests that the introduction of SiO_2_ does not alter the absorption bands of the organic ligands. Moreover, the coordination bond formed by SiO_2_ and Tm^3+^ can carry out more efficient energy transfer in thulium ions complexes molecules. Four samples with the strongest fluorescence spectra were detected at the excitation wavelength of 370 nm.

Figure 7c–f show the emission spectra of Tm(TTA/DBM)_3_phen and SiO_2_-Tm(TTA/DBM)_3_phen hybrid materials. In Figure 7c, the emission peak of SiO_2_-Tm(TTA)_3_phen is basically unchanged compared with Tm(TTA)_3_phen, while the fluorescence intensity is obviously enhanced. Tm(TTA)_3_phen and SiO_2_-Tm(TTA)_3_phen exhibit sharp peaks at 799 nm and 802 nm that are ascribed to ^3^H_4_→^3^H_6_ transitions of Tm^3+^ [28]. A wide emission peak appears at 1400 nm–1550 nm, which is caused by the ^3^H_4_→^3^F_4_ transitions of Tm^3+^. The emission peak of SiO_2_-Tm(TTA)_3_phen is higher and wider than that of Tm(TTA)_3_phen [29,30,31]. The highest intensity of Tm(TTA)_3_phen in the curve is 1387 (a.u.), while the highest intensity of SiO_2_-Tm(TTA)_3_phen in the curve is 2630 (a.u.), thus there is an enhancement about two times compared with pure complex; the data are given in Table 1. When HDBM is introduced as the ligand of trivalent thulium ion, the highest intensity of Tm(DBM)_3_phen in the curve is 1235 (a.u.), while the highest intensity of SiO_2_-Tm(DBM)_3_phen in the curve is 1280 (a.u.). Thus, there is also an enhancement compared with pure complex; the data are also shown in Table 1. In summary, the NIR-fluorescent hybrid materials have stronger fluorescence after combination with nano-SiO_2_ compared with pure Tm^3+^ complexes, and SiO_2_-Tm(TTA)_3_phen is more fluorescent than SiO_2_-Tm(DBM)_3_phen [32].

In addition, from Figure 7e, we can see the emission peak position of SiO_2_-Tm(DBM)_3_phen is basically unchanged compared with Tm(DBM)_3_phen, but it is different from Figure 7c; that is, the emission intensity of SiO_2_-Tm(DBM)_3_phen near 800 nm is slightly higher than that of Tm(DBM)_3_phen. The emission peak of SiO_2_-Tm(DBM)_3_phen at 1243 nm is originally the emission peak of Tm(DBM)_3_phen at 971nm. The fluorescence intensity of SiO_2_-Tm(DBM)_3_phen near 1400–1500 nm is higher than Tm(DBM)_3_phen. Figure 7d,f show that the fluorescence intensity of both Tm(DBM)_3_phen and SiO_2_-Tm(DBM)_3_phen is much lower than that of Tm(TTA)_3_phen and SiO_2_-Tm(TTA)_3_phen.

The UV–vis absorption spectra of Tm(TTA)_3_phen and Tm(DBM)_3_phen are shown in Figure S1. It can be found that the absorption peak of Tm(TTA)_3_phen and Tm(DBM)_3_phen is the strongest near 370 nm. According to the ultraviolet absorption spectrum analysis, the strong absorption capacity of HTTA/HDBM, and the high energy transfer efficiency of excited state in the ultraviolet region, HTTA/HDBM can help shorten the transition state of thulium ion in the ultraviolet region and improve the absorption coefficient of thulium ion in the ultraviolet region. The absorptive bandwidth of Tm(TTA)_3_phen is wider than that of Tm(DBM)_3_phen, combined with UV–vis absorption spectra shown in Figure S1 of Tm(DBM)_3_phen and Tm(TTA)_3_phen, which indicates that HTTA absorbs energy and delivers it more efficiently to Tm^3+^, resulting in better sensitized radiation of Tm^3+^. Therefore, HTTA has a better matching degree with Tm^3+^ than HDBM, which also indicates that Tm(TTA)_3_phen has a higher fluorescence yield than Tm(DBM)_3_phen.

The increase of fluorescence intensity of SiO_2_-Tm^3+^ hybrid materials is due to the carrying effect of SiO_2_-Tm^3+^. The amorphous nano-SiO_2_ provide a microenvironment for Tm^3+^ complex to limit energy consumption by reducing non-radiative transitions, leading to an increase in the fluorescence intensity of SiO_2_-Tm^3+^. According to the analysis of fluorescence spectra(Figure 7) and ultraviolet absorption spectrum(Figure S1), we find that Tm(TTA)_3_phen and SiO_2_-Tm(TTA)_3_phen hybrid nanoparticles have higher fluorescence than those of Tm(DBM)_3_phen and SiO_2_-Tm(DBM)_3_phen. These results show that HTTA is a better choice of organic ligands for Tm^3+^, and SiO_2_-Tm(TTA)_3_phen still has better fluorescence after the combination with nano-SiO_2_.

To further investigate the fluorescence properties of thulium ion complexes, the room-temperature fluorescence decay curves were measured in Figure S2, excited at 370 nm, and monitored at 802 nm. The decay curve of SiO_2_-Tm(TTA)_3_phen fits a single exponential function: D(t) = c_0_exp(−t/τ), with a lifetime of 50.38 μs.

**Figure 8:**
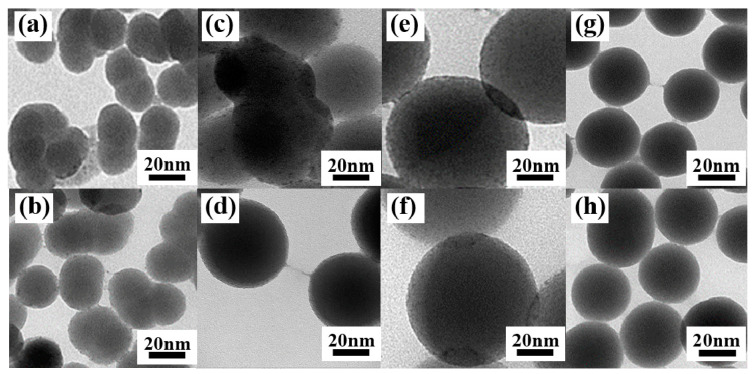
SiO_2_-Tm(DBM)_3_phen with a diameter of (**a**) 25 nm, (**c**) 50 nm, (**e**) 80 nm, and (**g**) 40 nm; SiO_2_-Tm(TTA)_3_phen with a diameter of (**b**) 25 nm, (**d**) 50 nm, (**f**) 80 nm, and (**h**) 40 nm.

Figure 8 shows the TEM images of SiO_2_-Tm^3+^ hybrid fluorescent materials with different particle size prepared under different NH_3_·H_2_O conditions. The measurements show that, when the dosage of NH_3_·H_2_O is increased from 1 mL to 3 mL, the diameters of SiO_2_-Tm^3+^ nanospheres are gradually increased and the dispersibility of the nanospheres are improved. Moreover, as can be seen in Figure 8, when the diameter of the SiO_2_-Tm^3+^ nanoparticles is 80 nm, the morphology of the hybrid fluorescent materials is uniform and maintains good dispersity. More Tm^3+^ complexes are attached to the nano-SiO_2_ nanoparticles in this case.

**Figure 9:**
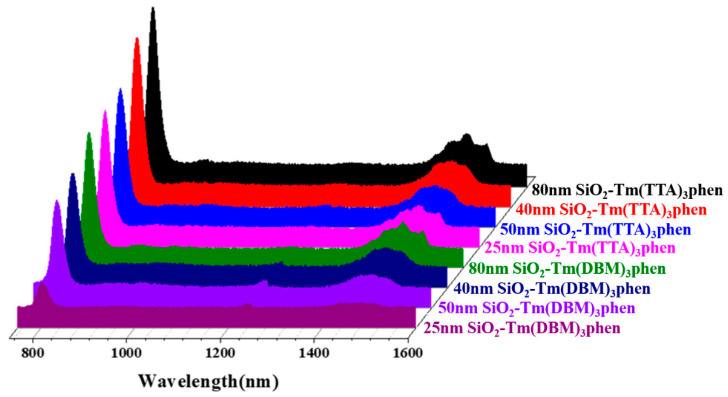
Fluorescence spectra of SiO_2_-Tm(DBM)_3_phen and SiO_2_-Tm(TTA)_3_phen with different diameters.

The fluorescence spectra of SiO_2_-Tm^3+^ hybrid fluorescent materials (SiO_2_-Tm(TTA)_3_phen and SiO_2_-Tm(DBM)_3_phen) of different particle sizes are shown in Figure 9. Of the four different sizes of SiO_2_-Tm(TTA)_3_phen, when the diameter of nanoparticles is 80 nm, the fluorescence intensity of SiO_2_-Tm(TTA)_3_phen is the highest. Similarly, of the four different sizes of SiO_2_-Tm(DBM)_3_phen, when the diameter of nanoparticles is 80 nm, the fluorescence intensity of SiO_2_-Tm(DBM)_3_phen is the highest. However, the fluorescence intensity of SiO_2_-Tm^3+^ hybrid fluorescent material with a diameter of 40 nm is higher than that of SiO_2_-Tm^3+^ hybrid fluorescent material with a diameter of 50 nm. This is because, when the content of NH_3_·H_2_O is high, the hydrolysis process of TEOS is accelerated. As the nano-SiO_2_ with a diameter of 40 nm has better dispersion, more Tm(TTA)_3_phen complexes get attached to each nano-SiO_2_ and embedded in the nanospheres in samples of the same volume. This gives higher fluorescence intensity.

## 4. Conclusions

In summary, SiO_2_-Tm(TTA/DBM)_3_phen NIR-fluorescent hybrid materials were prepared by an improved sol–gel method. By comparing the thulium ion complexes of two different organic ligands, we found that Tm(TTA)_3_phen has better fluorescent performance than that of Tm(DBM)_3_phen. In addition, the hybrid materials ofSiO_2_-Tm(TTA)_3_phen have better structural stability and thermal stability than that of SiO_2_-Tm(DBM)_3_phen. Moreover, fluorescence intensity of SiO_2_-Tm(TTA)_3_phen at 802 nm, 1231 nm, and 1400–1500 nm is significantly higher than that of Tm(TTA)_3_phen, Tm(DBM)_3_phen, and SiO_2_-Tm(DBM)_3_phen. Tm(TTA)_3_phen and SiO_2_-Tm(TTA)_3_phendisplay better fluorescent performance than that of Tm(DBM)_3_phen and SiO_2_-Tm(DBM)_3_phen. Therefore, HTTA is a better choice of organic ligand for Tm^3+^, and SiO_2_-Tm(TTA)_3_phen still has better fluorescent performance after the combination with nano-SiO_2_. In addition, the fluorescence intensity of SiO_2_-Tm(TTA/DBM)_3_phen of different sizes was studied, and it was found that it was the highest when the diameter of SiO_2_-Tm(TTA/DBM)_3_phen was 80 nm. We believe this kind of near-infrared fluorescent hybrid material with a more stable structure and stronger fluorescence will have prospects for food detection, biological detection, and biological imaging.

## Figures and Tables

**Table 1:** Enhancement of fluorescence intensity of Tm^3+^ complexes and SiO_2_-Tm^3+^ hybrid materials.

	Transitions	^3^H_4_→^3^H_6_ (a.u.)		^3^H_4_→^3^F_4_ (a.u.)	
Samples					
SiO_2_-Tm(TTA)_3_phen		2629	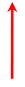	1049	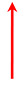
Tm(TTA)_3_phen		1396		884	
SiO_2_-Tm(DBM)_3_phen		1280		882	
Tm(DBM)_3_phen		1235		758	

## Supplementary Materials

The following are available online at https://www.mdpi.com/2079-4991/10/10/1964/s1, Figure S1: UV–vis absorption spectra of (a) Tm(DBM)3phen and (b) Tm(TTA)3phen, Figure S2: Fluorescence decay curve of SiO_2_-Tm(tta)_3_phen when excited at 370 nm and monitored at 803 nm.
