# Supplementary material for: NIR-Fluorescent Hybrid Materials of Tm3+ Complexes Carried by Nano-SiO2 via Improved Sol–Gel Method
Source: Nanomaterials (Basel). 2020 Oct 3;10(10):1964. doi: 10.3390/nano10101964 (PMC7600681; doi:10.3390/nano10101964)
Supplement: Supplementary file 1 [file nanomaterials-10-01964-s001.pdf]

nanomaterials

MDPI

Supporting information

NIR-Fluorescent Hybrid Materials of Tm^{3+} Complexes Carried by Nano-SiO_2 via Improved Sol–Gel Method

Yanxin Wang[1,*], Qiuyu Sun[1], Linjun Huang[1,*], Peng Lu[1], Xiaozhen Wang[1], Zhe Zhang[1],Yao Wang[1], JianguoTang[1,*], and Laurence A. Belfiore[2]

[1] Institute of Hybrid Materials, National Center of International Joint Research for Hybrid Materials Technology, National Base of International Sci. & Tech. Cooperation on Hybrid Materials, College of Materials Science and Engineering, Qingdao University, 308 Ningxia Road, Qingdao 266071, China; SQY17861431023@163.com (Q.S.); 18753360989@163.com (P.L.); wangxiaozhen686868@163.com (X.W.); 18363995370@163.com (Z.Z.); wangyaoqdu@126.com(Y.W.)

[2] Department of Chemical and Biological Engineering,Colorado State University, Fort Collins, CO 80523, USA; laurence.belfiore@colostate.edu

* Correspondence: wangyanxin@qdu.edu.cn (Y.W.); huanglinjun@qdu.edu.cn (L.H.); tang@qdu.edu.cn (J.T.)

Received: 22 September 2020; Accepted: 26 September 2020; Published: date

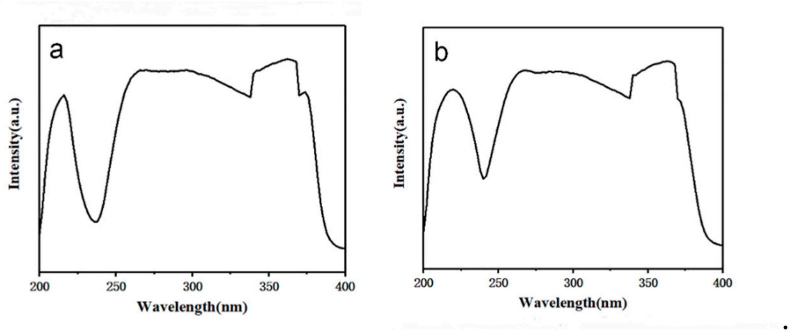

Figure S1. UV–vis absorption spectra of (a) Tm(DBM)$_3$phen and (b) Tm(TTA)$_3$phen.

Fig.S1 shows the ultraviolet absorption spectra of Tm(TTA)$_3$phen and Tm(DBM)$_3$phen. It can be found that the absorption peak of Tm(TTA)$_3$phen and Tm(DBM)$_3$phen is the strongest near 370nm. Therefore, according to the ultraviolet absorption spectrum analysis, due to the strong absorption capacity of HTTA/HDBM and the higher energy transfer efficiency of excited state in the ultraviolet region, HTTA/HDBM can help shorten the transition state of thulium ions in the ultraviolet region and improve the absorption coefficient of thulium ions in the ultraviolet region. However, the absorptive bandwidth of Tm(TTA)$_3$phen is wider than that of Tm(DBM)$_3$phen, which indicates that HTTA absorbs energy and delivers it more efficiently to Tm^{3+}, resulting in better desensitized radiation of Tm^{3+}. Therefore, Htta has a better matching degree with Tm^{3+} than HDBM, which also proves that Tm(TTA)$_3$phen has a better fluorescence performance than Tm(DBM)$_3$phen.

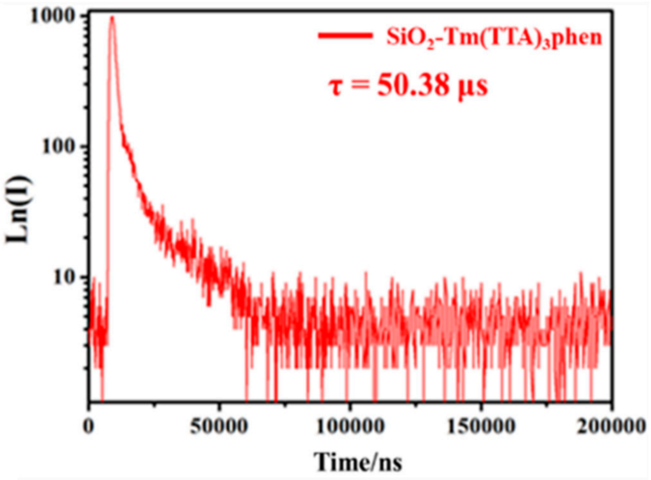

Figure S2. Fluorescence decay curve of SiO_2-Tm(tta)$_3$phen when excited at 370nm and monitored at 803nm. (the sample used for testing is solid; liquid nitrogen refrigeration temperature: -80℃.).

Fluorescence lifetime were measured by Edinburgh FLS-1000 steady-state transient fluorescence spectrometer (Edinburgh Inc., Livingston, UK) (342W Xe lamp). The decay curve of SiO_2-Tm(TTA)$_3$phen fits a single exponential function: $D(t) = c_0 exp(-t/\tau)$, the emission lifetime of SiO_2-Tm(TTA)$_3$phen is 50.38μs.
